# The Angelina Jolie effect: Contralateral risk-reducing mastectomy trends in patients at increased risk of breast cancer

**DOI:** 10.1038/s41598-021-82654-x

**Published:** 2021-02-02

**Authors:** Narendra Nath Basu, James Hodson, Shaunak Chatterjee, Ashu Gandhi, Julie Wisely, James Harvey, Lyndsey Highton, John Murphy, Nicola Barnes, Richard Johnson, Lester Barr, Cliona C. Kirwan, Sacha Howell, Andrew D. Baildam, Anthony Howell, D. Gareth Evans

**Affiliations:** 1 University of Birmingham, Birmingham, B15 2TT UK; 2 University Hospital Birmingham NHS Trust, Mindelsohn Way, Birmingham, B15 2TH UK; 3 Prevent Breast Cancer Research Unit, Wythenshawe Hospital, Manchester University NHS Foundation Trust, Manchester, M23 9LT UK; 4 Manchester Breast Centre, The Christie Hospital, Manchester, M20 UK; 5 NW Genomic Laboratory Hub, Manchester Centre for Genomic Medicine, Manchester University Hospitals NHS Foundation Trust, Manchester, M13 9WL UK; 6 Division of Evolution and Genomic Sciences, School of Biological Sciences, Faculty of Biology, Medicine and Health, University of Manchester, Manchester Academic Health Science Centre, Manchester, UK; 7 Clinical Genetics Service, Manchester Centre for Genomic Medicine, Manchester University Hospitals NHS Foundation Trust, Manchester, M13 9WL UK

**Keywords:** Cancer, Breast cancer

## Abstract

Contralateral risk-reducing mastectomy (CRRM) rates have tripled over the last 2 decades. Reasons for this are multi-factorial, with those harbouring a pathogenic variant in the *BRCA1*/*2* gene having the greatest survival benefit. On May 14th, 2013, Angelina Jolie shared the news of her bilateral risk-reducing mastectomy (BRRM), on the basis of her *BRCA1* pathogenic variant status. We evaluated the impact of this news on rates of CRRM in women with increased risk for developing breast cancer after being diagnosed with unilateral breast cancer. The prospective cohort study included all women with at least a moderate lifetime risk of developing breast cancer who attended our family history clinic (1987–2019) and were subsequently diagnosed with unilateral breast cancer. Rates of CRRM were then compared between patients diagnosed with breast cancer before and after Angelina Jolie’s announcement (pre- vs. post-AJ). Of 386 breast cancer patients, with a mean age at diagnosis of 48 ± 8 years, 268 (69.4%) were diagnosed in the pre-AJ period, and 118 (30.6%) in the post-AJ period. Of these, 123 (31.9%) underwent CRRM, a median 42 (interquartile range: 11–54) days after the index cancer surgery. Rates of CRRM doubled following AJ’s news, from 23.9% pre-AJ to 50.0% post AJ (*p* < 0.001). Rates of CRRM were found to decrease with increasing age at breast cancer (*p* < 0.001) and tumour TNM stage (*p* = 0.040), and to increase with the estimated lifetime risk of breast cancer (*p* < 0.001) and tumour grade (*p* = 0.015) on univariable analysis. After adjusting for these factors, the step-change increase in CRRM rates post-AJ remained significant (odds ratio: 9.61, *p* < 0.001). The AJ effect appears to have been associated with higher rates of CRRM amongst breast cancer patients with increased cancer risk. CRRM rates were highest amongst younger women and those with the highest lifetime risk profile. Clinicians need to be aware of how media news can impact on the delivery of cancer related services. Communicating objective assessment of risk is important when counselling women on the merits of risk-reducing surgery.

## Introduction

In recent years, there has been an increase in the number of unilaterally affected breast cancer patients undergoing removal of the opposite healthy breast^1^—contralateral risk-reducing mastectomy (CRRM). Data from the US Surveillance, Epidemiology and End Results (SEER) program^2^ has shown a 300% increase in rates of CRRM (2002—3.9% to 2012—12.7%). This significant increase is surprising, given that overall rates of contralateral breast cancer are decreasing^3^, likely due to improved preventive effects of anti-endocrine therapy^4^, as well as there being no improvements in long term outcomes associated with CRRM in the majority of breast cancer patients^5^.

However, in women deemed to be at increased risk of developing breast cancer based on their family history or carrier status of a pathogenic genetic mutation (e.g. *BRCA1*/*2*), a survival benefit from CRRM has been shown, particularly in those with a pathogenic variant in *BRCA1* and *BRCA2*^6–8^ . This group of patients have an annual contralateral breast cancer (CBC) rate of approximately 2–3%^9^, compared to approximately 0.5% in patients with sporadic breast cancer^4^. Amongst high-risk patients who test negative for the *BRCA1* or *BRCA2* mutation, the risk of CBC appears to remain elevated, although some studies have suggested otherwise^10^.

On May 14th 2013, Angelina Jolie (AJ) shared with the world her experience of bilateral risk reducing mastectomies (BRRMs)^11^ based on her inheriting a maternally derived pathogenic variant mutation in the *BRCA1* gene. Since this revelation, numerous changes in healthcare practices have been reported—the so-called “Angelina Jolie Effect”^12^. These include a 250% increase in referrals of women with a family history of breast cancer in the UK^13^, increases in rates of BRRM^14^ and health insurance reimbursement for *BRCA* testing^15–17^. In 2015, Angelina Jolie wrote about her experience with risk reducing salpingo-oophorectomy^18^—the effect of this on risk-reducing gynaecological surgery is yet to be determined.

Healthcare professionals need to be aware of the impact high profile personalities can have on health related issues. In 1987, Nancy Reagan’s well publicised decision to undergo a mastectomy^19^ over breast conservation in the USA resulted in a 25% reduction in the use of breast conservation, sparking a media frenzy with headlines claiming that such a personally publicised decision “set us back 10 years”^20^.

The reasons for choosing a CRRM are complex—patient’s personal experience, family history, radiological imaging, tumour factors, surgeon related factors and desire for symmetry/cosmesis need to be considered^21, 22^. In addition, breast cancer patients may substantially overestimate their risk, possibly influenced by the fact that some healthcare professionals have a poor understanding of CBC risk themselves^23, 24^. A challenging clinical scenario for breast clinicians is the newly diagnosed breast cancer patient who requests a CRRM. An understanding of the multiple factors discussed is critical to ensure this group of women receive evidence-based recommendations^25, 26^.

We analysed the trends of CRRM over a 29-year period amongst increased risk patients diagnosed with a unilateral breast cancer. Tumour and patient characteristics were assessed. The impact, if any, of the news of Angelina Jolie’s BRRM and her *BRCA* status was examined. To our knowledge, this is the first study to assess whether the “Angelina Jolie effect” affected the rate of CRRM amongst patients at an increased risk of developing a further breast cancer.

## Methods

Patients with an increased risk of developing breast cancer due to family history have been attending a dedicated Breast Cancer Family History Risk and Prevention Clinic (The Nightingale Breast Cancer Prevention Centre [NBCPC], Manchester, UK) for over 3 decades (1987- to date), with 14,198 women having been assessed as of February 2020. This dedicated service offers women appropriate risk assessment, radiological surveillance, lifestyle advice and risk-reducing strategies (BRRM, CRRM and chemoprevention). Lifetime and residual risk of breast cancer has been assessed using questionnaire information on family history and standard risk factors using Claus tables and Tyrer-Cuzick programme^27–29^. In the NBCPC, testing for *BRCA1* and *BRCA2* mutations has been available within our services since 1996. Additional genes are tested based on family history pedigree and cancer phenotype. Panel testing is not routinely performed. Ethics approval is covered by FHrisk Research Ethics Committee reference number 10/H1008/24.

The Family History database, a prospectively maintained database of all patients seen in the family history clinic was interrogated. All patients with a moderate or high risk of developing breast cancer (> 17% lifetime risk at any time during follow up)^30^ who were subsequently diagnosed with a unilateral breast cancer were included. Data relating to patient demographics and pathology, as per National Health Screening Breast Screening Programme guidelines^31^, were assessed.

The results of genetic testing and risk categorisation were also collected. However, data were only recorded for the most recent risk status; hence it was not known whether this information was available to the patient at the time the decisions regarding CRRM were made. Those patients who underwent CRRM were then identified, and rates were compared between those diagnosed with breast cancer in the periods before (pre-AJ) and after (post-AJ) Angelina Jolie’s announcement of BRRM (14th May 2013). As such, the pre-AJ group comprised of patients diagnosed with breast cancer between 14th Feb 1990–13th May 2013, and the post-AJ group between 14th May 2013–29th Nov 2019.

### Ethics approval

All the authors confirm that all experimental protocols were approved the University of Manchester and the University of Birmingham. All methods were carried out in accordance with relevant guidelines and regulations set by the University of Manchester and the University of Birmingham. Informed consent was obtained from all subjects (no subjects under 18) in accordance to the Ethics Committee at Manchester University NHS Foundation Trust. Our study protocols were covered by the Family History Risk application that gained ethics approval by the North West 7 Research Ethics Committee (10/H10008/24).

## Statistical methods

Comparisons between patients diagnosed with breast cancer in the periods pre- and post-AJ were performed using independent samples t-tests for normally distributed variables, Mann–Whitney U tests for ordinal variables, and Fisher’s exact tests for nominal variables.

The change in the rate of CRRM post-AJ was initially analysed using a Fisher’s exact test to compare between the two periods. Trends over time were then assessed using a segmented regression approach. Prior to this analysis, we calculated two variables, the first stating the number of years from the AJ date to the date of the index cancer diagnosis (i.e. negative values for those diagnosed pre-AJ), and the second being an indicator variable, indicating whether the diagnosis was pre- or post-AJ. A binary logistic regression model was then produced, with these two factors and an interaction term as covariates, giving the following model structure:

$$\upbeta _{0} +\upbeta _{{1}} *\left[ {{\text{Years\,post-AJ}}} \right] +\upbeta _{{2}} *\left[ {{\text{Indicator}}} \right] +\upbeta _{{3}} *\left[ {{\text{Years\,post-AJ}}} \right]*\left[ {{\text{Indicator}}} \right]$$

where: Indicator = 0 if the diagnosis was pre-AJ, or 1 if post-AJ.

From this model, the “slope” in the proportion undergoing CRRM per year in the pre-AJ period could be estimated (β_1_), as could the step-change increase in the proportion undergoing CRRM in the period after the AJ announcement (β_2_). The coefficient for the interaction term (β_3_) then allowed for the slopes in CRRM rates to be compared between the pre- and post-AJ periods.

A multivariable binary logistic regression model was then produced, in order to identify independent predictors of CRRM. Due to the correlation between factors, a forwards stepwise approach was used to select only those that were independently associated with CRRM for inclusion in the model. The factors selected by the stepwise procedure were then entered into the previously described segmented regression model, to adjust for the effect of these potentially confounding factors. All analyses were performed using IBM SPSS 22 (IBM Corp. Armonk, NY), with *p* < 0.05 deemed to be indicative of statistical significance throughout.

## Results

### Patient demographics

A total of 387 women were diagnosed with unilateral breast cancer and met the inclusion criteria of the study, with the first diagnosis being made in February 1990. Of these, one patient underwent CRRM prior to breast cancer being diagnosed (the cancer was an incidental finding), and so was excluded. Of the N = 386 patients included in the analysis, 268 (69.4%) were diagnosed prior to 14th May 2013, and were classified as pre-AJ, with the remainder (N = 118, 30.6%) being post-AJ. Comparisons between these two cohorts (Table 1) found no differences in patient age at breast cancer, or in the tumour size, grade, TNM stage (AJCC) or pathology. However, the post-AJ cohort were found to have significantly higher risk tumours (*p* < 0.001), with 55.1% vs. 36.6% being in the 40 + % lifetime risk category, and 58.3% vs. 35.2% being high (Non-*BRCA)* lifetime risk. The post-AJ cohort was also more likely to be lymph node negative (81.5% vs. 70.2%, *p* = 0.038), and had different distributions of molecular subtypes (*p* = 0.002), with fewer cases of triple-negative disease (17.9% vs. 30.1%).

**Table 1:** Patient demographics.

	N	Overall	Pre-AJ	Post-AJ	*p* value
Age at breast cancer (years)	386	47.8 ± 8.0	47.7 ± 8.3	48.0 ± 7.2	0.813
**Estimated lifetime risk (%)**	386				0.001*
< 30%		122 (31.6%)	94 (35.1%)	28 (23.7%)	
30–39%		101 (26.2%)	76 (28.4%)	25 (21.2%)	
40+%		163 (42.2%)	98 (36.6%)	65 (55.1%)	
**Risk category**	385				** < 0.001**
Moderate		117 (30.4%)	89 (33.3%)	28 (23.7%)	
High (non-BRCA)		163 (42.3%)	94 (35.2%)	69 (58.5%)	
BRCA1		54 (14.0%)	43 (16.1%)	11 (9.3%)	
BRCA2		50 (13.0%)	40 (15.0%)	10 (8.5%)	
BRCA1 and BRCA2		1 (0.3%)	1 (0.4%)	0 (0.0%)	
**Pathology**	381				0.729
IDC		276 (72.4%)	191 (72.1%)	85 (73.3%)	
ILC		30 (7.9%)	20 (7.5%)	10 (8.6%)	
Mixed IC		4 (1.0%)	4 (1.5%)	0 (0.0%)	
Pre-Invasive		71 (18.6%)	50 (18.9%)	21 (18.1%)	
**Size category**	367				0.432
Invasive: 0–19 mm		212 (57.8%)	152 (60.3%)	60 (52.2%)	
Invasive: 20+ mm		92 (25.1%)	58 (23.0%)	34 (29.6%)	
In-situ: 0–19 mm		39 (10.6%)	25 (9.9%)	14 (12.2%)	
In-situ: 20 mm		24 (6.5%)	17 (6.7%)	7 (6.1%)	
**Grade**	378				0.616
Grade 1		44 (11.6%)	31 (11.8%)	13 (11.2%)	
Grade 2		119 (31.5%)	77 (29.4%)	42 (36.2%)	
Grade 3		144 (38.1%)	104 (39.7%)	40 (34.5%)	
Carcinoma in-situ		71 (18.8%)	50 (19.1%)	21 (18.1%)	
**Lymph node status (pathological)**	323				0.038*
Negative		239 (74.0%)	151 (70.2%)	88 (81.5%)	
1–3 Nodes Positive		72 (22.3%)	56 (26.0%)	16 (14.8%)	
> 3 Nodes Positive		12 (3.7%)	8 (3.7%)	4 (3.7%)	
**TNM stage**	379				0.709*
Stage 0		68 (17.9%)	48 (18.2%)	20 (17.4%)	
Stage 1		188 (49.6%)	132 (50.0%)	56 (48.7%)	
Stage 2		107 (28.2%)	73 (27.7%)	34 (29.6%)	
Stage 3		16 (4.2%)	11 (4.2%)	5 (4.3%)	
**Molecular subtype**	343				**0.002**
Luminal A		238 (69.4%)	153 (67.7%)	85 (72.6%)	
Luminal B		10 (2.9%)	4 (1.8%)	6 (5.1%)	
Triple Negative		89 (25.9%)	68 (30.1%)	21 (17.9%)	
HER2 Enriched		6 (1.7%)	1 (0.4%)	5 (4.3%)	

Data are reported as N (column %), with *p* values from Fisher’s exact tests, or as mean ± SD, with *p* values from independent samples t-tests, unless stated otherwise. Bold *p* values are significant at *p* < 0.05. *p value from Mann–Whitney U test, as the factor is ordinal. *IDC* invasive ductal carcinoma, *ILC* invasive lobular carcinoma, *IC* invasive carcinoma.

### Rates of CRRM

In total, 31.9% (N = 123) of patients underwent CRRM, which was performed a median of 42 days (interquartile range: 11–154) after the index cancer surgery. Of those undergoing CRRM, this was performed during the index cancer surgery in 16.5% of cases, with the longest interval between breast cancer and CRRM being 58 months, and intervals being less than 28 months for the remainder. There were three patients diagnosed with breast cancer prior to the AJ announcement, and underwent CRRM in the post-AJ period, who were included in the pre-AJ group for analysis. However, in two of these patients, the interval from breast cancer to CRRM was less than three months; hence the decision to have CRRM was likely made prior to the AJ announcement. The third patient had an interval of 10 months between breast cancer and CRRM.

### Change over time in CRRM rates

**Figure 1:**
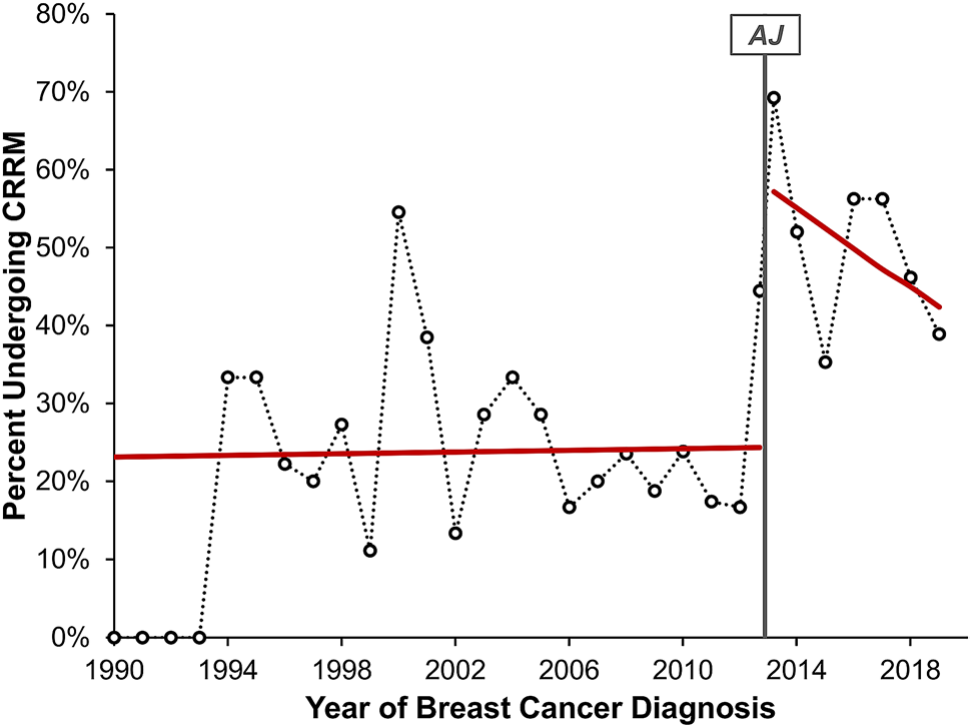
Segmented regression analysis of CRRM. Points represent the observed rate of CRRM in each year of the study, with the exception of 2013, which is divided into the period pre- and post-AJ. The red line represents the segmented binary logistic regression model reported in Table 2, whilst the grey line indicates the timing of the AJ announcement.

A simple comparison between the pre- and post-AJ periods found a significant difference in rates of CRRM (*p* < 0.001), with a two-fold increase from 23.9% (64/268) to 50.0% (59/118). For the subgroup of *BRCA1*/*2* carriers (N = 105), a similar increase in CRRM rates from 47.6% (40/84) to 90.5% (19/21) was observed between the periods (*p* < 0.001). The trends in CRRMs were further assessed using a segmented regression approach, the results of which are summarised in Table 2 and Fig. 1. In the pre-AJ period, the rate of CRRMs was not found to be changing over time [odds ratio (OR): 1.00 per year, *p* = 0.907]. Immediately after the AJ announcement, a significant step change increase in the CRRM rate was observed (OR: 4.29, *p* < 0.001). In the post-AJ period, the rates of CRRM were not found to be changing significantly over time (OR: 0.90 per year, *p* = 0.267), although there appeared to be a tendency for a decline.

**Table 2:** Segmented regression analysis of CRRM.

Component	Odds ratio (95% CI)	*p* value
Pre-AJ gradient (per year)	1.00 (0.95–1.05)	0.907
Step change	4.29 (1.84–10.0)	**< 0.001**
Change in gradient post-AJ (per year)*	0.90 (0.74–1.09)	0.271

Results are from a segmented binary logistic regression model, as described in the methods. Bold *p* values are significant at *p* < 0.05. *Represents the difference between the gradients in the pre- vs. post-AJ periods. Re-parameterisation of the model found the gradient in the post-AJ period to have an odds ratio of 0.90 per year (95% CI 0.75–1.08, *p* = 0.267).

### Other factors associated with CRRM

**Figure 2:**
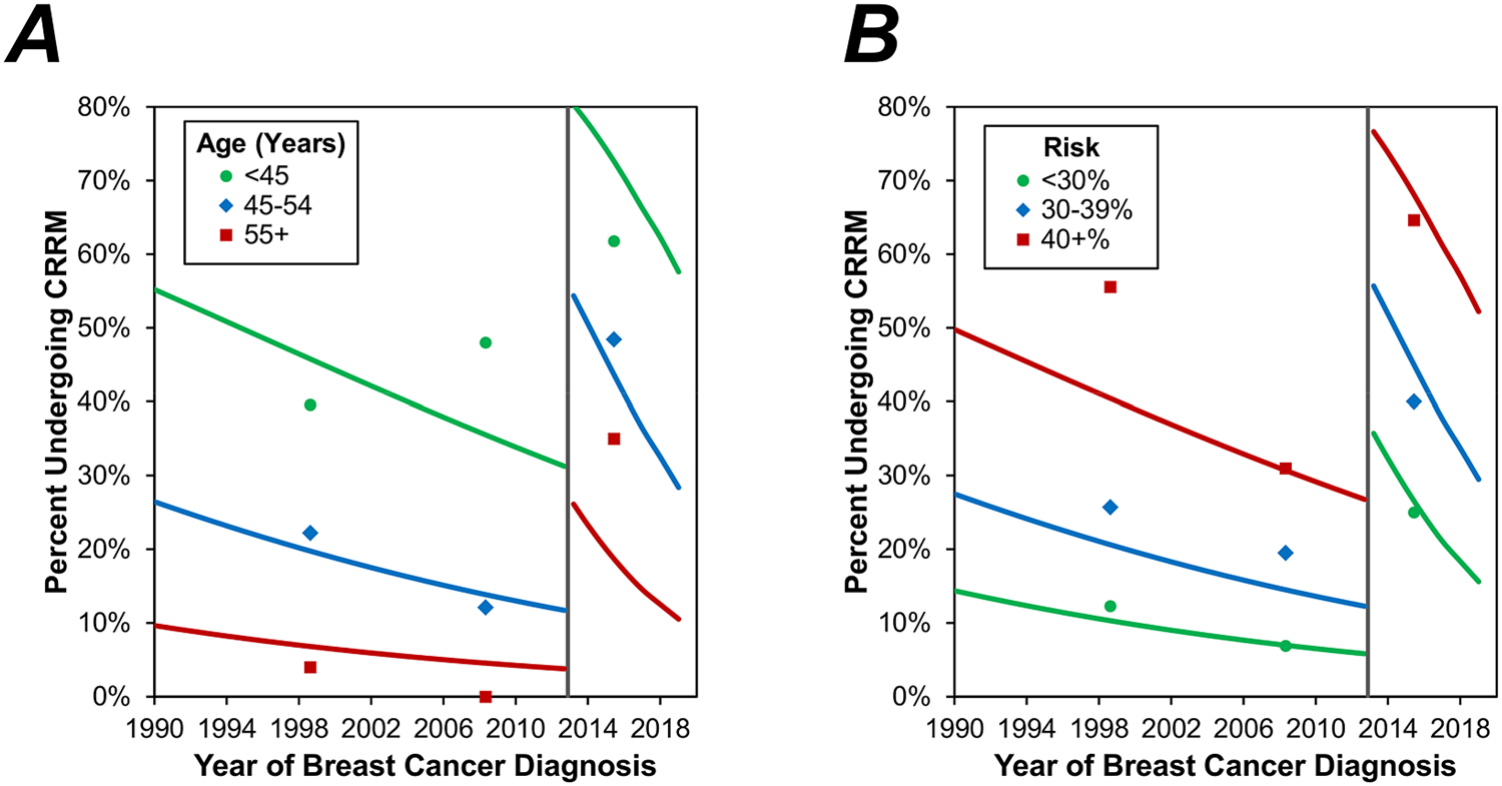
Multivariable analysis of CRRM by age and estimated lifetime risk. Points represent the observed rate of CRRM in three periods (1990–2004, 2005-AJ, AJ-2019), and are plotted at the midpoint of the interval. Trend lines represent the multivariable model reported in Table 4, which was evaluated within each of the age and estimated lifetime risk categories, respectively. These trend lines are for the “average” patient; hence (**A**) is evaluated based on the distribution of risk categories from Table 1, and (**B**) based on an age of 47.8 years. The grey line indicates the timing of the AJ announcement.

Across the cohort as a whole, rates of CRRM were found to decrease significantly with increasing age at breast cancer (*p* < 0.001) and TNM tumour stage (*p* = 0.040), and to increase significantly with risk (estimate lifetime/category, both *p* < 0.001) and tumour grade (*p* = 0.015) on univariable analysis (Table 3). A multivariable analysis was then performed, in order to assess the changes over time in CRRM rates, after accounting for these potentially confounding factors (Table 4, Fig. 2). This identified younger age and higher estimated lifetime risk to be significant independent predictors of CRRM (both *p* < 0.001). After adjusting for these factors, the step-change increase in CRRM rates post-AJ remained significant (OR: 9.61, *p* < 0.001). However, the subsequent decline in the CRRM rate was found to be more pronounced on multivariable analysis, with a near-significant OR of 0.83 per year (*p* = 0.076) in the post-AJ period.

**Table 3:** Associations between patient factors and CRRM rates.

	N	CRRM rate	*p* value
**Age at breast cancer (years)**			< 0.001*
< 45	132	64 (48.5%)	
45–49	109	33 (30.3%)	
50–54	75	18 (24.0%)	
55 +	70	8 (11.4%)	
**Estimated lifetime risk (%)**			< 0.001*
< 30%	122	17 (13.9%)	
30–39%	101	27 (26.7%)	
40+%	163	79 (48.5%)	
Risk category**			**< 0.001**
Moderate	117	17 (14.5%)	
High (non-BRCA)	163	47 (28.8%)	
BRCA1	54	29 (53.7%)	
BRCA2	50	30 (60.0%)	
**Pathology**			0.509
IDC	276	84 (30.4%)	
ILC	30	9 (30.0%)	
Mixed IC	4	1 (25.0%)	
Pre-Invasive	71	28 (39.4%)	
**Size category**			0.793
Invasive: 0–19 mm	212	64 (30.2%)	
Invasive: 20+ mm	92	29 (31.5%)	
In-situ: 0–19 mm	39	14 (35.9%)	
In-situ: 20 mm	24	9 (37.5%)	
**Grade**			**0.015**
Grade 1	44	6 (13.6%)	
Grade 2	119	36 (30.3%)	
Grade 3	144	52 (36.1%)	
Carcinoma in-situ	71	28 (39.4%)	
**Lymph node status (pathological)**			0.196*
Negative	239	77 (32.2%)	
1–3 nodes Positive	72	19 (26.4%)	
> 3 nodes Positive	12	2 (16.7%)	
**TNM stage**			0.040*
Stage 0	68	28 (41.2%)	
Stage 1	188	61 (32.4%)	
Stage 2	107	30 (28.0%)	
Stage 3	16	3 (18.8%)	
**Molecular subtype**			0.327
Luminal A	238	72 (30.3%)	
Luminal B	10	2 (20.0%)	
Triple Negative	89	34 (38.2%)	
HER2 enriched	6	3 (50.0%)	

Data are reported as N (row %), with *p* values from Fisher’s exact tests, unless stated otherwise. Bold *p* values are significant at *p* < 0.05. *p value from Mann–Whitney U test, as the factor is ordinal. **Excludes N = 1 with both BRCA1 and BRCA2 mutations. *IDC* invasive ductal carcinoma, *ILC* Invasive Lobular carcinoma, *IC* invasive carcinoma.

**Table 4:** Multivariable analysis of CRRM.

	Odds ratio (95% CI)	*p* value
AJ to cancer (years)	0.96 (0.90–1.02)	0.170
Step-change	9.61 (3.54–26.14)	**< 0.001**
Change in gradient post-AJ (per year)*	0.86 (0.69–1.08)	0.189
Age at breast cancer (per decade)	0.30 (0.20–0.43)	**< 0.001**
**Estimated lifetime risk (%)**		**< 0.001**
< 30%	–	**–**
30–39%	2.26 (1.08–4.75)	**0.031**
40+%	5.90 (2.97–11.73)	**< 0.001**

Results are from a multivariable binary logistic regression model. All factors in Table 1 were initially considered for inclusion in the model, with forwards stepwise approach used to select those that were independently associated with CRRM. Factors selected by the stepwise procedure were then entered into a model alongside the factors from Table 2. Bold *p* values are significant at *p* < 0.05. *Represents the difference between the gradients in the pre- vs. post-AJ periods. Re-parameterisation of the model found the gradient in the post-AJ period to have an odds ratio of 0.83 per year (95% CI 0.67–1.02, *p* = 0.076).

## Discussion

Our study demonstrates that in women diagnosed with unilateral breast cancer, attending a family history clinic, rates of CRRM in the 20 years prior to AJ were stable, at approximately 24%. This rate more than doubled to 51.8% following the AJ announcement, with a sustained longer-term effect four years later.

These observed trends in the pre-AJ period are in contrast to overall trends from the UK (based on hospital episode statistics)^32^ and the US^1^, where CRRM rates over this period have doubled and tripled , respectively. It is important to emphasise that all patients in our study had an elevated CBC risk, based on family history or mutation status, whereas the aforementioned studies were based on SEER data^1^ and the Hospital Episode Statistics (HES) database, where data on risk was not available. A study from Switzerland^33^ showed no change in the rates of CRRM over time, and concluded that differences in medico-social and cultural factors, perceptions towards breast cancer and access to plastic surgery may account for differences between the US and Europe.

There are a number of studies that have assessed the impact of genetic testing on CRRM rates^34–36^. A recent study from Denmark^35^ confirmed that uptake of CRRM amongst women before the age of 45 years was significantly higher in those harbouring a pathogenic variant of the *BRCA1*/*2* gene and those from high-risk families, compared to those with a lower genetic risk.

To our knowledge, this is the first study to report variations in rates of CRRM according to baseline lifetime risk of developing breast cancer. Patients attending the family history clinic are likely to have differing motivations for CRRM, compared to those with a sporadic cancer, although this has not been formally assessed. In addition, international variations exist towards attitudes to risk-reducing surgery. For example, in Poland, only 2.7% of *BRCA* carriers chose a BRRM, compared to 36% in the US^37^, suggesting cultural factors and access to breast reconstruction may contribute to this.

In our study, CRRM was most commonly performed in younger breast cancer patients (*p* < 0.001) who, amongst *BRCA1* and *BRCA2* carriers, derive the greatest survival benefit^6–8^. Tuttle et al.^1^ found that favourable prognostic factors (low tumour grade, negative nodal status and small tumour size) were associated with increasing rates of CRRM. They proposed that this was because this group of patients with a good breast cancer prognosis were likely to derive the most benefit from CRRM, as “their survival time is longer and thus their subsequent risk of contralateral breast cancer is greater”. These factors were not found to be significant independent predictors of CRRM in our study, although our cohort reflects breast cancers in the context of early detection, with later stage breast cancers being uncommon. In addition, on account of the relatively small sample size, it is possible that the study was underpowered to detect the associations between these factors and CRRM.

Overall uptake of CRRM in our cohort was 31.9% over the study period as a whole. There are wide variations in uptake of CRRM worldwide with the highest for *BRCA1*/*2* carriers in North America (49.3%), compared to 0% in Norway^38^. Our own studies suggest that attitudes towards risk reducing surgery amongst breast surgeons in the UK are closely aligned with the USA^39^. As expected, this rate was highest amongst the youngest (< 45 years) and those with the highest risk profile (40 + % estimated lifetime risk), which is in keeping with previous studies^38^.

We recently reported an increase in the number of high-risk women undergoing BRRM following the Angelina Jolie’s’ article in the New York Times in May 2013^14^. There was an up to six month delay in this increase—as women undergoing BRRM (in comparison to CRRM) follow a time consuming detailed protocol, including psychological assessment^40^. This is in contrast to CRRM, where the rise in number was almost immediate, suggesting that surgeons and multi-disciplinary teams may be willing to consider CRRM as part of the primary treatment, rather than undertaking a lengthier protocol similar to women being considered for BRRM. In addition, the effect seems to be sustained over a period of at least four years, although there is some decline in number of CRRMs following the initial surge. Long term follow up will be important to assess whether rates of CRRMs ultimately return to the pre-AJ baseline and, if so, how long the AJ effect persisted.

Our study demonstrated that almost a fifth of patients undergoing CRRM did so on the basis of a Stage 0 (DCIS—ductal carcinoma in situ) index breast cancer. This group with pre-invasive breast cancer is considered to have excellent long-term outcomes following breast conserving surgery or mastectomy. Mastectomy rates for DCIS declined between 1998 and 2004, with rates increasing to 2011^41^. In the USA, women being diagnosed with DCIS are increasingly choosing bilateral mastectomies^41^, with a recent study^42^ showing that, despite adjusting for family history, rates of CBC amongst those diagnosed with DCIS are not increased sufficiently to warrant a recommendation of CRRM. Further work needs to determine the current trend of CRRM amongst women with DCIS.

Amongst women who tested positive for a pathogenic variant in either *BRCA* genes, uptake of CRRM in the post-AJ period was extremely high, at 90%. Data from a recent prospective study^43^ assessing the effect of a germline *BRCA1* or *BRCA2* mutation on breast cancer outcomes in patients with young-onset breast cancer found that this group (tested positive for *BRCA1* or *BRCA2*) had similar survival compared to non-carriers. Therefore, clinicians need to be aware that this group of high-risk patients warrant further consideration when discussing risk-reducing strategies.

There are some limitations to the current study. We have reported the results from a single centre; hence the generalizability of findings to other centres and other countries cannot be confirmed. This also resulted in a relatively small sample size, although this did yield sufficient statistical power to obtain high levels of significance when analysing the AJ effect, even across risk groups. A high proportion of cases underwent genetic testing, allowing sub-analysis based on carrier status. However, the details of the genetic testing was not available to all patients prior to CRRM, hence may not have directly influenced their decision. Whilst there appeared to be a tendency for CRRM rates to be in decline after the initial step-change increase, the relatively short period of follow up in the post-AJ period was insufficient to conclusively assess whether this decline was significant. In addition, in the later years of the study, it is possible that rates of CRRM may have been underestimated, as patients may have opted to undergo CRRM after the follow up period had ended. This may be more pronounced in those undergoing neoadjuvant chemotherapy, which may delay the primary surgical decision, and may be particularly relevant in *BRCA1*, carriers whose tumours nearly always require chemotherapy. However, the times from surgery to CRRM were generally short, with a median of 42 days, hence this should have had minimal influence on the results.

We have previously reviewed the multiple risk factors for developing contralateral breast cancers and potential drivers for CRRM^4^. It has not been possible to adjust for these multiple factors in our current study. However, access to breast reconstruction has remained constant over the period of the study. Despite this, there the type of breast reconstruction (autologous or implant based) may be a confounding factors, although data were not available to assess this. In addition, access to neoadjuvant chemotherapy, changing trends in MRI and both patient and physician knowledge on objective risk assessment are other potentially important confounding factors for which data were not available to be assessed in the current study. . In addition to these factors that it was not possible to adjust for, it is possible that residual confounding may exist for factors that were accounted for in the multivariable analysis, particularly those that are correlated with both the outcome (CRRM) and primary factor (AJ), such as the estimated lifetime risk. As such, the results of the multivariable analyses should be interpreted in the light of the limitations of both unmeasured variables and residual confounding.

We recently published the “Manchester Guidelines for CRRM”^25^, which proposed a protocol to be used in the decision-making process regarding CBC, based on a review of the literature. The current study furthers our understanding into potential drivers for variations in rates of CRRM, highlighting that highly publicised case-studies of CRRM may have a sizable influence on patient decision making. In light of this, it is important to ensure that objective assessments of risk are clearly communicated when counselling women on the merits of risk-reducing surgery, to ensure that they have sufficient information to make a rational decision, and are not unduly influenced by high-profile cases.

## Conclusion

The “Angelina Jolie effect” appears to be have been associated with a sustained increase in the rates of CRRM amongst increased risk breast cancer patients in our single-centre study. When considering the trends over time of CRRM, it may be important to assess those with a significant family history and/or those harbouring a pathogenic variant in one of the breast cancer susceptibility genes as a separate group to those with sporadic breast cancer. Further studies should address the multiple factors that contribute to a decision for CRRM.

## Data Availability

The datasets generated and/or analysed during the current study are not publicly available as this contains patient sensitive material. It may be available from the corresponding author on reasonable request.

## Abbreviations

CRRM: Contralateral risk reducing mastectomy
BRRM: Bilateral risk reducing mastectomy
DCIS: Ductal carcinoma in situ
ILC: Invasive lobular carcinoma
IDC: Invasive ductal carcinoma
AJ: Angelina Jolie
CBC: Contralateral breast cancer
TNM: Classification of malignant tumours (tumour node metastasis)
